# Crystal structure of *trans*-(1,8-dibutyl-1,3,6,8,10,13-hexa­aza­cyclo­tetra­decane-κ^4^ *N* ^3^,*N* ^6^,*N* ^10^,*N* ^13^)bis­(thio­cyanato-κ*N*)nickel(II) from synchrotron data

**DOI:** 10.1107/S205698901501110X

**Published:** 2015-06-13

**Authors:** Dae-Woong Kim, Jong Jin Kim, Jong Won Shin, Jin Hong Kim, Dohyun Moon

**Affiliations:** a Department of Chemistry and Green-Nano Materials Research Center, Kyungpook National University, Daegu, 702-701, Republic of Korea; b Department of Applied Chemistry, College of Engineering, Kyungpook National University, Daegu, 702-701, Republic of Korea; c Beamline Department, Pohang Accelerator Laboratory 80, Jigokro-127-beongil, Nam-Gu Pohang, Gyeongbuk 790-784, South Korea

**Keywords:** crystal structure, aza­macrocyclic ligand, Jahn–Teller distortion, sodium thio­cyanate, hydrogen bonding, synchrotron data

## Abstract

The crystal structure of the title compound, [Ni(NCS)_2_(C_16_H_38_N_6_)], has been determined from synchrotron data. The asymmetric unit consists of two halves of the complex mol­ecules which have their Ni^II^ atoms located on inversion centres. The Ni^II^ ions show a tetra­gonally distorted octa­hedral coordination geometry, with four secondary amine N atoms of the aza­macrocyclic ligand in the equatorial plane and two N atoms of the thio­cyanate anions in the axial positions. The average equatorial Ni—N bond length [2.070 (5) Å] is shorter than the average axial Ni—N bond length [2.107 (18) Å]. Only half of the macrocyclic ligand N—H groups are involved in hydrogen bonding. The complex mol­ecules are connected *via* inter­molecular N—H⋯S hydrogen bonds into two symmetry-independent one-dimensional polymeric structures extending along the *b-*axis direction. One of the *n*-butyl substituents of the macrocycle exhibits conformational disorder with a refined occupancy ratio of 0.630:0.370.

## Abstract 2

The Ni^II^ atom in the title compound, bonded to four N atoms of the aza­macrocylic ligand and two N atoms of the thio­cyanate ions, shows a slightly distorted octa­hedral coordination geometry. In the crystal, mol­ecules are connected by hydrogen bonds, forming chains along the *b-*axis direction.

## Chemical context   

Coordination compounds, including those formed by macrocyclic ligands, have attracted wide inter­est of material sciences, because of their potential applications (Lehn, 1995 ▸; Zhou *et al.*, 2012 ▸). In particular, Ni^II^ macrocyclic complexes having vacant sites in the axial positions have been used for the synthesis of new supra­molecular materials with inter­esting properties, including chiral recognition (Ryoo *et al.*, 2010 ▸) and gas storage (Suh *et al.*, 2012 ▸). For example, Ni^II^ complexes with alkyl-substituted tetra-aza­macrocyclic ligands and anionic tetra­zole derivatives, metal cyanide and azide (Shen *et al.*, 2012 ▸; Kim *et al.*, 2015 ▸) have been studied as magnetic materials and substrates for crystal engineering. The thio­cyanate ion is a versatile anionic ligand which can easily bind to a transition metal ion as a terminal or bridging ligand through the nitro­gen and/or the sulfur atoms, thus allowing the assembly of multi-dimensional compounds or heterometallic complexes (Safarifard & Morsali, 2012 ▸; Wang & Wang, 2015 ▸). Here, we report the synthesis and crystal structure of an Ni^II^ complex with an aza­macrocycle ligand and two thio­cyanate anions, *trans*-(1,8-dibutyl-1,3,6,8,10,13-hexa­aza­cyclo­tetra­decane-*κ^4^N* ^3^ *,N* ^6^ *,N* ^10^ *,N* ^13^)bis(thio­cyanato*-κN*)nickel(II) (**I**).
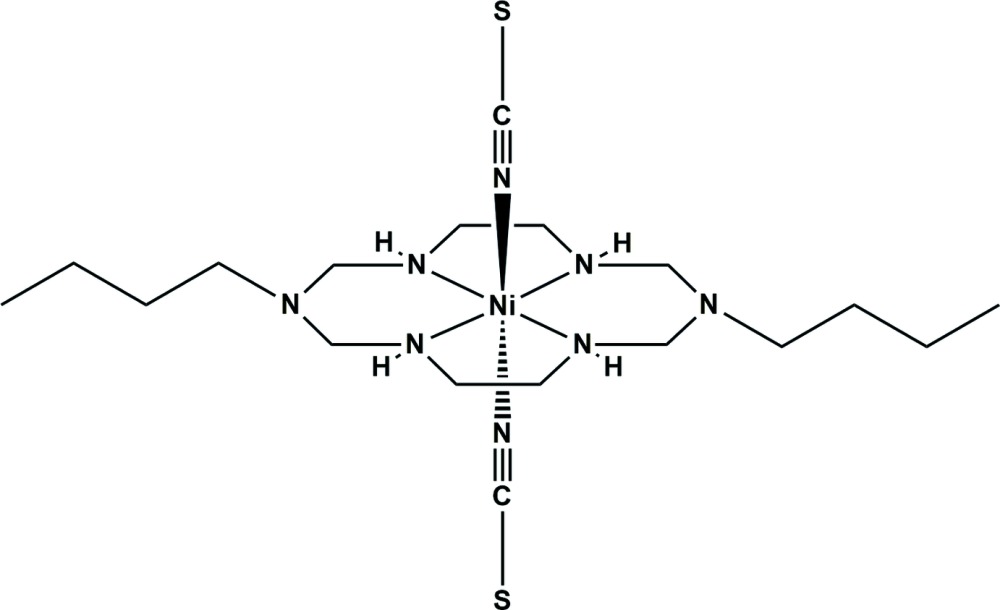


## Structural commentary   

**Figure 1:**
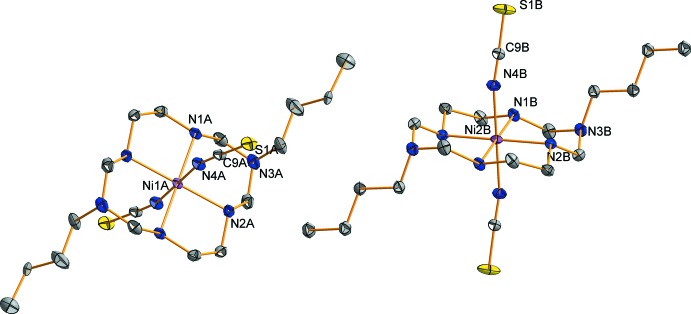
View of the mol­ecular structure of the title compound, showing the atom-labelling scheme, with displacement ellipsoids drawn at the 30% probability level. H atoms have been omitted for clarity. The minor position of the *n*-butyl substituent in the *A* mol­ecule is not shown.

The title compound (**I**) contains two crystallographically independent complex mol­ecules that are centrosymmetric. Each Ni^II^ ion lies on an inversion centre and is coordinated by four secondary amine N atoms of the aza­macrocyclic ligand in a square-planar fashion in the equatorial plane, and by two N atoms from the thio­cyanate anions at the axial positions, resulting in a tetra­gonally distorted octa­hedral geometry, as shown in Fig. 1 ▸. The average equatorial bond lengths, Ni1*A*—N_eq_ and Ni1*B*—N_eq_, are 2.070 (8) and 2.070 (3) Å, respectively. The axial bond lengths, Ni1*A*—N_ax_ and Ni1*B*—N_ax_ are 2.119 (1) and 2.093 (1) Å, respectively. The axial bonds are longer than the equatorial bonds, which can be attributed either to a large Jahn–Teller distortion effect of the Ni^II^ ion and/or to a ring contraction of the aza­macrocyclic ligand (Halcrow, 2013 ▸; Kim *et al.*, 2015 ▸). The average N—C and C—S bond lengths of the thiocyanate ligands are 1.157 (1) and 1.627 (11) Å, respectively. The former is very similar to a C N triple-bond length, while the latter is slightly shorter than reported C—S single-bond lengths (Bradforth *et al.*, 1993 ▸; Shin *et al.*, 2010 ▸). The six-membered chelate rings involving C2*A*, C3*A* and C2*B*, C3*B* atoms adopt a *chair* conformation, whereas the five-membered chelate rings involving C1*A*, C4*A* and C1*B*, C4*B* assume a *gauche* conformation (Min & Suh, 2001 ▸; Kim *et al.*, 2015 ▸).

## Supra­molecular features   

**Figure 2:**
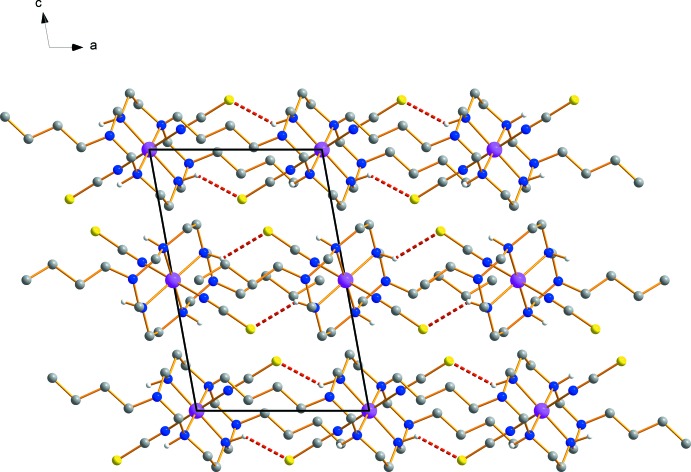
View of the crystal packing, with N—H⋯S hydrogen bonds drawn as red dashed lines. H atoms have been omitted for clarity.

The S atoms of the thio­cyanate groups form inter­molecular N—H⋯S hydrogen bonds with adjacent secondary amine groups of the aza­macrocyclic ligand, giving rise to two symmetry-independent one-dimensional polymeric chains propagating along the *b*-axis direction (Fig. 2 ▸ and Table 1 ▸).

## Database survey   

A search of the Cambridge Structural Database (Version 5.36, Feb 2015 with two updates; Groom & Allen, 2014 ▸) indicated one complex of Ni^II^ with the same aza­maclocyclic ligand having an anionic tetra­zole derivative at the axial positions (Kim *et al.*, 2015 ▸).

## Synthesis and crystallization   

The title compound (**I**) was prepared as follows. The starting complex, [Ni(C_16_H_38_N_6_)](ClO_4_)_2_, was prepared by a slightly modified method reported by Jung *et al.* (1989 ▸). To a MeCN solution (10 mL) of [Ni(C_16_H_38_N_6_)](ClO_4_)_2_ (0.15 g, 0.26 mmol) was slowly added a MeCN solution (5 mL) containing sodium thio­cyanate (0.042 g, 0.52 mmol) at room temperature. A pale-pink precipitate was formed, which was filtered off, washed with MeCN, and diethyl ether, and dried in air. Single crystals of the title compound were obtained by layering of the MeCN solution of sodium thio­cyanate on the MeCN solution of [Ni(C_16_H_38_N_6_)](ClO_4_)_2_ for several days. Yield: 0.062 g (49%). FT–IR (KBr, cm^−1^): 3304, 3243, 2929, 2867, 2069, 1468, 1386, 1273, 1204, 1070, 925.

**Safety note:** Although we have experienced no problem with the compounds reported in this study, perchlorate salts of metal complexes are often explosive and should be handled with great caution.

## Refinement   

Crystal data, data collection and structure refinement details are summarized in Table 2 ▸. All H atoms were placed in geometrically idealized positions and constrained to ride on their parent atoms, with C—H distances of 0.98–0.99 Å and an N–H distance of 1.0 Å with *U*_iso_(H) values of 1.2 or 1.5*U*_eq_ of the parent atoms. The C7*A* and C8*A* atoms of the macrocyclic ligand were refined as disordered over two sets of sites (C71*A*, C72*A* and C81*A*, C82*A*) with refined occupancies of 0.630 and 0.370, respectively. The bond lengths and angles of the disordered part were restrained to ensure proper geometry using DFIX and DANG instructions of *SHELXL2014* (Sheldrick, 2015*b* ▸).

## Supplementary Material

Crystal structure: contains datablock(s) I. DOI: 10.1107/S205698901501110X/gk2635sup1.cif

Structure factors: contains datablock(s) I. DOI: 10.1107/S205698901501110X/gk2635Isup2.hkl

CCDC reference: 1405450

Additional supporting information:  crystallographic information; 3D view; checkCIF report

## Figures and Tables

**Table 1:** Hydrogen-bond geometry (, )

*D*H*A*	*D*H	H*A*	*D* *A*	*D*H*A*
N1*A*H1*A*S1*A* ^i^	1.00	2.73	3.5154(17)	136
N2*B*H2*B*S1*B* ^ii^	1.00	2.66	3.4556(17)	137

Symmetry codes: (i) 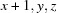; (ii) 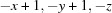.

**Table 2:** Experimental details

Crystal data	
Chemical formula	[Ni(NCS)_2_(C_16_H_38_N_6_)]
*M*_r_	489.39
Crystal system, space group	Triclinic, *P* 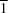
Temperature (K)	180
*a*, *b*, *c* ()	8.6610(17), 12.027(2), 12.560(3)
, , ()	94.66(3), 97.99(3), 110.04(3)
*V* (^3^)	1205.4(5)
*Z*	2
Radiation type	Synchrotron, = 0.630
(mm^1^)	0.72
Crystal size (mm)	0.25 0.15 0.13

Data collection	
Diffractometer	ADSC Q210 CCD area detector
Absorption correction	Empirical (using intensity measurements) (*HKL3000sm *SCALEPACK**; Otwinowski Minor, 1997 ▸)
*T*_min_, *T*_max_	0.841, 0.916
No. of measured, independent and observed [*I* > 2(*I*)] reflections	12812, 6583, 6243
*R*_int_	0.014
(sin /)_max_ (^1^)	0.696

Refinement	
*R*[*F* ^2^ > 2(*F* ^2^)], *wR*(*F* ^2^), *S*	0.042, 0.111, 1.06
No. of reflections	6583
No. of parameters	287
No. of restraints	11
H-atom treatment	H-atom parameters constrained
_max_, _min_ (e ^3^)	1.58, 1.11

Computer programs: *PAL ADSC Quantum-210 ADX* (Arvai Nielsen, 1983 ▸), *HKL3000sm* (Otwinowski Minor, 1997 ▸), *SHELXT2014* (Sheldrick, 2015*a* ▸), *SHELXL2014* (Sheldrick, 2015*b* ▸), *DIAMOND* (Putz Brandenburg, 2007 ▸) and *publCIF* (Westrip, 2010 ▸).

## supplementary crystallographic information

### Crystal data

**Table:**

[Ni(NCS)_2_(C_16_H_38_N_6_)]	*Z* = 2
*M**_r_* = 489.39	*F*(000) = 524
Triclinic, *P*1	*D*_x_ = 1.348 Mg m^−^^3^
*a* = 8.6610 (17) Å	Synchrotron radiation, λ = 0.630 Å
*b* = 12.027 (2) Å	Cell parameters from 49914 reflections
*c* = 12.560 (3) Å	θ = 0.4–33.6°
α = 94.66 (3)°	µ = 0.72 mm^−^^1^
β = 97.99 (3)°	*T* = 180 K
γ = 110.04 (3)°	Block, pale pink
*V* = 1205.4 (5) Å^3^	0.25 × 0.15 × 0.13 mm

### Data collection

**Table:**

ADSC Q210 CCD area-detector diffractometer	6243 reflections with *I* > 2σ(*I*)
Radiation source: PLSII 2D bending magnet	*R*_int_ = 0.014
ω scan	θ_max_ = 26.0°, θ_min_ = 1.6°
Absorption correction: empirical (using intensity measurements) (*HKL3000sm *SCALEPACK**; Otwinowski & Minor, 1997)	*h* = −12→12
*T*_min_ = 0.841, *T*_max_ = 0.916	*k* = −16→16
12812 measured reflections	*l* = −17→17
6583 independent reflections	

### Refinement

**Table:**

Refinement on *F*^2^	11 restraints
Least-squares matrix: full	Hydrogen site location: inferred from neighbouring sites
*R*[*F*^2^ > 2σ(*F*^2^)] = 0.042	H-atom parameters constrained
*wR*(*F*^2^) = 0.111	*w* = 1/[σ^2^(*F*_o_^2^) + (0.0541*P*)^2^ + 0.7946*P*] where *P* = (*F*_o_^2^ + 2*F*_c_^2^)/3
*S* = 1.06	(Δ/σ)_max_ = 0.002
6583 reflections	Δρ_max_ = 1.58 e Å^−^^3^
287 parameters	Δρ_min_ = −1.11 e Å^−^^3^

### Special details

**Table:**

Geometry. All e.s.d.'s (except the e.s.d. in the dihedral angle between two l.s. planes) are estimated using the full covariance matrix. The cell e.s.d.'s are taken into account individually in the estimation of e.s.d.'s in distances, angles and torsion angles; correlations between e.s.d.'s in cell parameters are only used when they are defined by crystal symmetry. An approximate (isotropic) treatment of cell e.s.d.'s is used for estimating e.s.d.'s involving l.s. planes.

### Fractional atomic coordinates and isotropic or equivalent isotropic displacement parameters (Å^2^)

**Table:**

	*x*	*y*	*z*	*U*_iso_*/*U*_eq_	Occ. (<1)
Ni1A	0.0000	0.0000	0.5000	0.02295 (8)	
S1A	−0.39772 (6)	0.09973 (6)	0.68745 (4)	0.05126 (15)	
N1A	0.20720 (17)	0.11708 (13)	0.60541 (11)	0.0301 (3)	
H1A	0.3083	0.1069	0.5828	0.036*	
N2A	0.02319 (18)	0.10693 (13)	0.37714 (12)	0.0321 (3)	
H2A	0.1105	0.0955	0.3377	0.039*	
N3A	0.2318 (2)	0.27979 (15)	0.49597 (17)	0.0460 (4)	
N4A	−0.16012 (18)	0.07346 (14)	0.56843 (13)	0.0343 (3)	
C1A	0.1944 (2)	0.07739 (18)	0.71320 (14)	0.0373 (4)	
H1A1	0.1128	0.1036	0.7459	0.045*	
H1A2	0.3042	0.1129	0.7621	0.045*	
C2A	0.2242 (3)	0.24393 (17)	0.60232 (18)	0.0426 (4)	
H2A1	0.1280	0.2567	0.6285	0.051*	
H2A2	0.3269	0.2957	0.6528	0.051*	
C3A	0.0780 (3)	0.23584 (17)	0.41904 (19)	0.0431 (4)	
H3A1	0.0916	0.2821	0.3571	0.052*	
H3A2	−0.0106	0.2499	0.4539	0.052*	
C4A	−0.1381 (2)	0.05792 (19)	0.30124 (15)	0.0387 (4)	
H4A1	−0.1251	0.0872	0.2304	0.046*	
H4A2	−0.2223	0.0840	0.3307	0.046*	
C5A	0.3770 (3)	0.2784 (3)	0.4483 (3)	0.0632 (7)	
H5A1	0.3705	0.3095	0.3778	0.076*	
H5A2	0.3693	0.1945	0.4332	0.076*	
C6A	0.5445 (3)	0.3493 (3)	0.5164 (4)	0.0870 (11)	
H6A1	0.5622	0.3131	0.5829	0.104*	
H6A2	0.5534	0.4327	0.5378	0.104*	
C71A	0.6816 (5)	0.3457 (6)	0.4395 (4)	0.084 (2)	0.63
H71A	0.6781	0.2628	0.4233	0.100*	0.63
H71B	0.6563	0.3747	0.3701	0.100*	0.63
C81A	0.8493 (5)	0.4237 (4)	0.4995 (4)	0.0654 (11)	0.63
H81A	0.8483	0.5035	0.5213	0.098*	0.63
H81B	0.9324	0.4296	0.4525	0.098*	0.63
H81C	0.8780	0.3895	0.5642	0.098*	0.63
C72A	0.7095 (11)	0.3319 (9)	0.5052 (9)	0.077 (2)	0.37
H72A	0.8005	0.3782	0.5662	0.093*	0.37
H72B	0.6971	0.2465	0.4991	0.093*	0.37
C82A	0.7346 (11)	0.3790 (12)	0.4057 (7)	0.090 (3)	0.37
H82A	0.6498	0.3254	0.3461	0.135*	0.37
H82B	0.8460	0.3857	0.3921	0.135*	0.37
H82C	0.7255	0.4582	0.4107	0.135*	0.37
C9A	−0.2607 (2)	0.08368 (15)	0.61628 (13)	0.0304 (3)	
Ni2B	1.0000	0.5000	0.0000	0.02474 (8)	
S1B	0.48562 (7)	0.51503 (7)	−0.18976 (6)	0.0654 (2)	
N1B	0.90438 (18)	0.33335 (13)	−0.09357 (12)	0.0319 (3)	
H1B	0.7910	0.3226	−0.1338	0.038*	
N2B	0.86585 (17)	0.44340 (13)	0.12179 (11)	0.0293 (3)	
H2B	0.7493	0.4404	0.0979	0.035*	
N3B	0.7849 (2)	0.23013 (14)	0.05324 (14)	0.0368 (3)	
N4B	0.79629 (19)	0.53663 (16)	−0.07763 (14)	0.0384 (3)	
C1B	1.0161 (2)	0.34041 (17)	−0.17379 (16)	0.0395 (4)	
H1B1	0.9614	0.2749	−0.2350	0.047*	
H1B2	1.1210	0.3322	−0.1394	0.047*	
C2B	0.8871 (3)	0.23310 (16)	−0.02905 (18)	0.0401 (4)	
H2B1	0.8377	0.1568	−0.0789	0.048*	
H2B2	0.9997	0.2392	0.0065	0.048*	
C3B	0.8572 (2)	0.32257 (17)	0.14559 (15)	0.0363 (3)	
H3B1	0.9715	0.3258	0.1735	0.044*	
H3B2	0.7904	0.3008	0.2037	0.044*	
C4B	0.9457 (2)	0.53919 (17)	0.21515 (14)	0.0366 (4)	
H4B1	1.0503	0.5321	0.2515	0.044*	
H4B2	0.8700	0.5320	0.2685	0.044*	
C5B	0.6079 (2)	0.20371 (17)	0.00919 (15)	0.0366 (4)	
H5B1	0.5718	0.1390	−0.0530	0.044*	
H5B2	0.5956	0.2756	−0.0184	0.044*	
C6B	0.4936 (2)	0.16605 (17)	0.09165 (15)	0.0382 (4)	
H6B1	0.5143	0.0997	0.1253	0.046*	
H6B2	0.5206	0.2340	0.1498	0.046*	
C7B	0.3107 (3)	0.12633 (19)	0.04111 (16)	0.0413 (4)	
H7B1	0.2794	0.0511	−0.0089	0.050*	
H7B2	0.2932	0.1877	−0.0020	0.050*	
C8B	0.1979 (3)	0.1066 (2)	0.12585 (18)	0.0456 (4)	
H8B1	0.2212	0.0508	0.1725	0.068*	
H8B2	0.0808	0.0734	0.0895	0.068*	
H8B3	0.2190	0.1831	0.1701	0.068*	
C9B	0.6659 (2)	0.52684 (14)	−0.12328 (13)	0.0294 (3)	

### Atomic displacement parameters (Å^2^)

**Table:**

	*U*^11^	*U*^22^	*U*^33^	*U*^12^	*U*^13^	*U*^23^
Ni1A	0.02036 (13)	0.02643 (14)	0.02328 (13)	0.00930 (10)	0.00718 (9)	0.00057 (9)
S1A	0.0351 (2)	0.0888 (4)	0.0415 (3)	0.0343 (3)	0.01563 (19)	0.0052 (3)
N1A	0.0239 (6)	0.0347 (7)	0.0292 (6)	0.0085 (5)	0.0064 (5)	−0.0031 (5)
N2A	0.0294 (6)	0.0369 (7)	0.0333 (7)	0.0130 (5)	0.0111 (5)	0.0087 (5)
N3A	0.0402 (8)	0.0320 (7)	0.0611 (11)	0.0053 (6)	0.0126 (8)	0.0082 (7)
N4A	0.0292 (6)	0.0389 (7)	0.0376 (7)	0.0161 (6)	0.0098 (5)	−0.0021 (6)
C1A	0.0309 (8)	0.0535 (10)	0.0258 (7)	0.0150 (7)	0.0046 (6)	−0.0020 (7)
C2A	0.0391 (9)	0.0315 (8)	0.0492 (10)	0.0061 (7)	0.0071 (8)	−0.0078 (7)
C3A	0.0437 (10)	0.0344 (9)	0.0555 (11)	0.0155 (8)	0.0138 (8)	0.0144 (8)
C4A	0.0354 (8)	0.0551 (11)	0.0305 (8)	0.0204 (8)	0.0076 (6)	0.0118 (7)
C5A	0.0370 (11)	0.0618 (15)	0.0824 (18)	−0.0004 (10)	0.0211 (11)	0.0257 (13)
C6A	0.0414 (13)	0.0560 (16)	0.147 (3)	−0.0010 (11)	0.0047 (17)	0.0253 (19)
C71A	0.042 (2)	0.129 (4)	0.055 (2)	−0.011 (2)	0.0025 (17)	0.067 (3)
C81A	0.051 (2)	0.062 (2)	0.076 (3)	0.0168 (18)	0.0036 (19)	−0.003 (2)
C72A	0.062 (5)	0.074 (5)	0.098 (7)	0.026 (4)	0.016 (5)	0.019 (5)
C82A	0.083 (7)	0.129 (10)	0.054 (5)	0.049 (7)	−0.015 (5)	−0.011 (6)
C9A	0.0253 (7)	0.0374 (8)	0.0300 (7)	0.0144 (6)	0.0043 (5)	−0.0002 (6)
Ni2B	0.02097 (13)	0.02802 (14)	0.02616 (14)	0.01142 (10)	0.00207 (9)	0.00079 (10)
S1B	0.0371 (3)	0.0927 (5)	0.0605 (4)	0.0332 (3)	−0.0185 (2)	−0.0201 (3)
N1B	0.0280 (6)	0.0312 (6)	0.0350 (7)	0.0090 (5)	0.0090 (5)	−0.0017 (5)
N2B	0.0241 (6)	0.0333 (6)	0.0283 (6)	0.0093 (5)	0.0025 (5)	0.0009 (5)
N3B	0.0358 (7)	0.0314 (7)	0.0425 (8)	0.0102 (6)	0.0093 (6)	0.0065 (6)
N4B	0.0285 (7)	0.0482 (9)	0.0418 (8)	0.0189 (6)	0.0019 (6)	0.0083 (7)
C1B	0.0345 (8)	0.0373 (9)	0.0424 (9)	0.0079 (7)	0.0145 (7)	−0.0086 (7)
C2B	0.0419 (9)	0.0297 (8)	0.0524 (11)	0.0152 (7)	0.0153 (8)	0.0037 (7)
C3B	0.0341 (8)	0.0393 (9)	0.0347 (8)	0.0119 (7)	0.0039 (6)	0.0102 (7)
C4B	0.0299 (8)	0.0446 (9)	0.0289 (7)	0.0070 (7)	0.0060 (6)	−0.0040 (7)
C5B	0.0354 (8)	0.0332 (8)	0.0363 (8)	0.0054 (6)	0.0084 (7)	0.0042 (6)
C6B	0.0386 (9)	0.0371 (8)	0.0339 (8)	0.0067 (7)	0.0089 (7)	0.0046 (7)
C7B	0.0397 (9)	0.0445 (10)	0.0339 (8)	0.0069 (8)	0.0105 (7)	0.0029 (7)
C8B	0.0434 (10)	0.0489 (11)	0.0440 (10)	0.0125 (8)	0.0158 (8)	0.0083 (8)
C9B	0.0283 (7)	0.0303 (7)	0.0309 (7)	0.0131 (6)	0.0055 (6)	0.0003 (6)

### Geometric parameters (Å, º)

**Table:**

Ni1A—N1A^i^	2.0640 (17)	C82A—H82A	0.9800
Ni1A—N1A	2.0640 (17)	C82A—H82B	0.9800
Ni1A—N2A^i^	2.0754 (15)	C82A—H82C	0.9800
Ni1A—N2A	2.0754 (15)	Ni2B—N2B^ii^	2.0675 (15)
Ni1A—N4A^i^	2.1190 (15)	Ni2B—N2B	2.0675 (15)
Ni1A—N4A	2.1190 (15)	Ni2B—N1B^ii^	2.0719 (16)
S1A—C9A	1.6339 (17)	Ni2B—N1B	2.0719 (16)
N1A—C1A	1.478 (2)	Ni2B—N4B^ii^	2.0933 (16)
N1A—C2A	1.486 (2)	Ni2B—N4B	2.0933 (16)
N1A—H1A	1.0000	S1B—C9B	1.6190 (18)
N2A—C4A	1.477 (2)	N1B—C1B	1.479 (2)
N2A—C3A	1.483 (3)	N1B—C2B	1.484 (2)
N2A—H2A	1.0000	N1B—H1B	1.0000
N3A—C3A	1.436 (3)	N2B—C4B	1.480 (2)
N3A—C2A	1.440 (3)	N2B—C3B	1.486 (2)
N3A—C5A	1.470 (3)	N2B—H2B	1.0000
N4A—C9A	1.158 (2)	N3B—C3B	1.444 (3)
C1A—C4A^i^	1.517 (3)	N3B—C2B	1.446 (2)
C1A—H1A1	0.9900	N3B—C5B	1.469 (3)
C1A—H1A2	0.9900	N4B—C9B	1.156 (2)
C2A—H2A1	0.9900	C1B—C4B^ii^	1.523 (3)
C2A—H2A2	0.9900	C1B—H1B1	0.9900
C3A—H3A1	0.9900	C1B—H1B2	0.9900
C3A—H3A2	0.9900	C2B—H2B1	0.9900
C4A—C1A^i^	1.517 (3)	C2B—H2B2	0.9900
C4A—H4A1	0.9900	C3B—H3B1	0.9900
C4A—H4A2	0.9900	C3B—H3B2	0.9900
C5A—C6A	1.501 (4)	C4B—C1B^ii^	1.522 (3)
C5A—H5A1	0.9900	C4B—H4B1	0.9900
C5A—H5A2	0.9900	C4B—H4B2	0.9900
C6A—C72A	1.537 (9)	C5B—C6B	1.520 (3)
C6A—C71A	1.641 (6)	C5B—H5B1	0.9900
C6A—H6A1	0.9900	C5B—H5B2	0.9900
C6A—H6A2	0.9900	C6B—C7B	1.514 (3)
C71A—C81A	1.485 (5)	C6B—H6B1	0.9900
C71A—H71A	0.9900	C6B—H6B2	0.9900
C71A—H71B	0.9900	C7B—C8B	1.522 (3)
C81A—H81A	0.9800	C7B—H7B1	0.9900
C81A—H81B	0.9800	C7B—H7B2	0.9900
C81A—H81C	0.9800	C8B—H8B1	0.9800
C72A—C82A	1.427 (12)	C8B—H8B2	0.9800
C72A—H72A	0.9900	C8B—H8B3	0.9800
C72A—H72B	0.9900		
			
N1A^i^—Ni1A—N1A	180.00 (7)	C72A—C82A—H82B	109.5
N1A^i^—Ni1A—N2A^i^	95.00 (7)	H82A—C82A—H82B	109.5
N1A—Ni1A—N2A^i^	85.00 (6)	C72A—C82A—H82C	109.5
N1A^i^—Ni1A—N2A	85.00 (6)	H82A—C82A—H82C	109.5
N1A—Ni1A—N2A	95.00 (6)	H82B—C82A—H82C	109.5
N2A^i^—Ni1A—N2A	180.00 (8)	N4A—C9A—S1A	178.09 (16)
N1A^i^—Ni1A—N4A^i^	91.75 (6)	N2B^ii^—Ni2B—N2B	180.0
N1A—Ni1A—N4A^i^	88.25 (6)	N2B^ii^—Ni2B—N1B^ii^	93.91 (6)
N2A^i^—Ni1A—N4A^i^	92.85 (6)	N2B—Ni2B—N1B^ii^	86.09 (6)
N2A—Ni1A—N4A^i^	87.15 (6)	N2B^ii^—Ni2B—N1B	86.09 (6)
N1A^i^—Ni1A—N4A	88.25 (6)	N2B—Ni2B—N1B	93.91 (6)
N1A—Ni1A—N4A	91.75 (6)	N1B^ii^—Ni2B—N1B	180.0
N2A^i^—Ni1A—N4A	87.15 (6)	N2B^ii^—Ni2B—N4B^ii^	88.26 (6)
N2A—Ni1A—N4A	92.85 (6)	N2B—Ni2B—N4B^ii^	91.74 (6)
N4A^i^—Ni1A—N4A	180.0	N1B^ii^—Ni2B—N4B^ii^	88.42 (7)
C1A—N1A—C2A	114.56 (15)	N1B—Ni2B—N4B^ii^	91.58 (7)
C1A—N1A—Ni1A	106.14 (11)	N2B^ii^—Ni2B—N4B	91.74 (6)
C2A—N1A—Ni1A	112.51 (12)	N2B—Ni2B—N4B	88.26 (6)
C1A—N1A—H1A	107.8	N1B^ii^—Ni2B—N4B	91.58 (7)
C2A—N1A—H1A	107.8	N1B—Ni2B—N4B	88.42 (7)
Ni1A—N1A—H1A	107.8	N4B^ii^—Ni2B—N4B	180.0
C4A—N2A—C3A	115.13 (15)	C1B—N1B—C2B	114.04 (15)
C4A—N2A—Ni1A	105.93 (11)	C1B—N1B—Ni2B	104.88 (11)
C3A—N2A—Ni1A	112.70 (12)	C2B—N1B—Ni2B	113.56 (11)
C4A—N2A—H2A	107.6	C1B—N1B—H1B	108.0
C3A—N2A—H2A	107.6	C2B—N1B—H1B	108.0
Ni1A—N2A—H2A	107.6	Ni2B—N1B—H1B	108.0
C3A—N3A—C2A	116.46 (17)	C4B—N2B—C3B	114.37 (14)
C3A—N3A—C5A	113.3 (2)	C4B—N2B—Ni2B	104.95 (10)
C2A—N3A—C5A	116.6 (2)	C3B—N2B—Ni2B	112.98 (11)
C9A—N4A—Ni1A	161.18 (15)	C4B—N2B—H2B	108.1
N1A—C1A—C4A^i^	108.32 (14)	C3B—N2B—H2B	108.1
N1A—C1A—H1A1	110.0	Ni2B—N2B—H2B	108.1
C4A^i^—C1A—H1A1	110.0	C3B—N3B—C2B	115.91 (15)
N1A—C1A—H1A2	110.0	C3B—N3B—C5B	115.92 (16)
C4A^i^—C1A—H1A2	110.0	C2B—N3B—C5B	113.83 (16)
H1A1—C1A—H1A2	108.4	C9B—N4B—Ni2B	163.23 (16)
N3A—C2A—N1A	113.64 (16)	N1B—C1B—C4B^ii^	108.49 (15)
N3A—C2A—H2A1	108.8	N1B—C1B—H1B1	110.0
N1A—C2A—H2A1	108.8	C4B^ii^—C1B—H1B1	110.0
N3A—C2A—H2A2	108.8	N1B—C1B—H1B2	110.0
N1A—C2A—H2A2	108.8	C4B^ii^—C1B—H1B2	110.0
H2A1—C2A—H2A2	107.7	H1B1—C1B—H1B2	108.4
N3A—C3A—N2A	113.85 (16)	N3B—C2B—N1B	113.94 (15)
N3A—C3A—H3A1	108.8	N3B—C2B—H2B1	108.8
N2A—C3A—H3A1	108.8	N1B—C2B—H2B1	108.8
N3A—C3A—H3A2	108.8	N3B—C2B—H2B2	108.8
N2A—C3A—H3A2	108.8	N1B—C2B—H2B2	108.8
H3A1—C3A—H3A2	107.7	H2B1—C2B—H2B2	107.7
N2A—C4A—C1A^i^	108.23 (15)	N3B—C3B—N2B	114.19 (14)
N2A—C4A—H4A1	110.1	N3B—C3B—H3B1	108.7
C1A^i^—C4A—H4A1	110.1	N2B—C3B—H3B1	108.7
N2A—C4A—H4A2	110.1	N3B—C3B—H3B2	108.7
C1A^i^—C4A—H4A2	110.1	N2B—C3B—H3B2	108.7
H4A1—C4A—H4A2	108.4	H3B1—C3B—H3B2	107.6
N3A—C5A—C6A	115.5 (3)	N2B—C4B—C1B^ii^	108.66 (15)
N3A—C5A—H5A1	108.4	N2B—C4B—H4B1	110.0
C6A—C5A—H5A1	108.4	C1B^ii^—C4B—H4B1	110.0
N3A—C5A—H5A2	108.4	N2B—C4B—H4B2	110.0
C6A—C5A—H5A2	108.4	C1B^ii^—C4B—H4B2	110.0
H5A1—C5A—H5A2	107.5	H4B1—C4B—H4B2	108.3
C5A—C6A—C72A	125.6 (5)	N3B—C5B—C6B	113.59 (16)
C5A—C6A—C71A	105.5 (3)	N3B—C5B—H5B1	108.8
C5A—C6A—H6A1	110.6	C6B—C5B—H5B1	108.8
C71A—C6A—H6A1	110.6	N3B—C5B—H5B2	108.8
C5A—C6A—H6A2	110.6	C6B—C5B—H5B2	108.8
C71A—C6A—H6A2	110.6	H5B1—C5B—H5B2	107.7
H6A1—C6A—H6A2	108.8	C7B—C6B—C5B	112.38 (16)
C81A—C71A—C6A	107.8 (4)	C7B—C6B—H6B1	109.1
C81A—C71A—H71A	110.2	C5B—C6B—H6B1	109.1
C6A—C71A—H71A	110.2	C7B—C6B—H6B2	109.1
C81A—C71A—H71B	110.2	C5B—C6B—H6B2	109.1
C6A—C71A—H71B	110.2	H6B1—C6B—H6B2	107.9
H71A—C71A—H71B	108.5	C6B—C7B—C8B	112.29 (17)
C71A—C81A—H81A	109.5	C6B—C7B—H7B1	109.1
C71A—C81A—H81B	109.5	C8B—C7B—H7B1	109.1
H81A—C81A—H81B	109.5	C6B—C7B—H7B2	109.1
C71A—C81A—H81C	109.5	C8B—C7B—H7B2	109.1
H81A—C81A—H81C	109.5	H7B1—C7B—H7B2	107.9
H81B—C81A—H81C	109.5	C7B—C8B—H8B1	109.5
C82A—C72A—C6A	99.0 (7)	C7B—C8B—H8B2	109.5
C82A—C72A—H72A	112.0	H8B1—C8B—H8B2	109.5
C6A—C72A—H72A	112.0	C7B—C8B—H8B3	109.5
C82A—C72A—H72B	112.0	H8B1—C8B—H8B3	109.5
C6A—C72A—H72B	112.0	H8B2—C8B—H8B3	109.5
H72A—C72A—H72B	109.6	N4B—C9B—S1B	178.44 (17)
C72A—C82A—H82A	109.5		
			
C2A—N1A—C1A—C4A^i^	167.05 (14)	C5A—C6A—C72A—C82A	71.8 (8)
Ni1A—N1A—C1A—C4A^i^	42.27 (15)	C2B—N1B—C1B—C4B^ii^	−167.20 (15)
C3A—N3A—C2A—N1A	73.7 (2)	Ni2B—N1B—C1B—C4B^ii^	−42.36 (16)
C5A—N3A—C2A—N1A	−64.4 (2)	C3B—N3B—C2B—N1B	−71.3 (2)
C1A—N1A—C2A—N3A	−178.39 (15)	C5B—N3B—C2B—N1B	66.9 (2)
Ni1A—N1A—C2A—N3A	−57.03 (18)	C1B—N1B—C2B—N3B	176.89 (15)
C2A—N3A—C3A—N2A	−73.1 (2)	Ni2B—N1B—C2B—N3B	56.80 (19)
C5A—N3A—C3A—N2A	66.4 (2)	C2B—N3B—C3B—N2B	72.1 (2)
C4A—N2A—C3A—N3A	177.61 (16)	C5B—N3B—C3B—N2B	−65.2 (2)
Ni1A—N2A—C3A—N3A	55.96 (19)	C4B—N2B—C3B—N3B	−177.77 (14)
C3A—N2A—C4A—C1A^i^	−167.20 (15)	Ni2B—N2B—C3B—N3B	−57.79 (17)
Ni1A—N2A—C4A—C1A^i^	−41.95 (15)	C3B—N2B—C4B—C1B^ii^	166.62 (14)
C3A—N3A—C5A—C6A	166.0 (2)	Ni2B—N2B—C4B—C1B^ii^	42.25 (15)
C2A—N3A—C5A—C6A	−54.7 (3)	C3B—N3B—C5B—C6B	−58.2 (2)
N3A—C5A—C6A—C72A	159.1 (5)	C2B—N3B—C5B—C6B	163.62 (16)
N3A—C5A—C6A—C71A	−173.6 (3)	N3B—C5B—C6B—C7B	−173.67 (16)
C5A—C6A—C71A—C81A	175.0 (4)	C5B—C6B—C7B—C8B	−171.39 (18)

Symmetry codes: (i) −*x*, −*y*, −*z*+1; (ii) −*x*+2, −*y*+1, −*z*.

### Hydrogen-bond geometry (Å, º)

**Table:**

*D*—H···*A*	*D*—H	H···*A*	*D*···*A*	*D*—H···*A*
N1*A*—H1*A*···S1*A*^iii^	1.00	2.73	3.5154 (17)	136
N2*B*—H2*B*···S1*B*^iv^	1.00	2.66	3.4556 (17)	137

Symmetry codes: (iii) *x*+1, *y*, *z*; (iv) −*x*+1, −*y*+1, −*z*.
